# Exciting Cause of Hydrophobia

**Published:** 1898-09

**Authors:** 


Exciting Cause of Hydrophobia. Dr. Grigorjew believes (*Centralblatt f. Bacteriologie*) that the exciting cause of hydrophobia is not a bacterium, but a body belonging to the protozoa. He has isolated from animals suffering from rabies a body with slow amœboid movements and exhibiting extension of pseudopods. Its action may even be modified by the presence of bacteria.

